# LncRNA‐HOTAIR promotes endothelial cell pyroptosis by regulating the miR‐22/NLRP3 axis in hyperuricaemia

**DOI:** 10.1111/jcmm.16812

**Published:** 2021-07-22

**Authors:** Kun Chi, Xiaodong Geng, Chao Liu, Yang Zhang, Jie Cui, GuangYan Cai, Xiangmei Chen, Fangfang Wang, Quan Hong

**Affiliations:** ^1^ Department of Nephrology Chinese PLA General Hospital Medical School of Chinese PLA Chinese PLA Institute of Nephrology State Key Laboratory of Kidney Diseases National Clinical Research Center for Kidney Diseases Beijing Key Laboratory of Kidney Diseases Beijing China; ^2^ Beidaihe Rehabilitation and Recuperation Center Chinese People’s Liberation Army Joint Logistics Support Force Qinhuangdao Qinhuangdao China; ^3^ Department of Cardiology and Institute of Vascular Medicine NHC Key Laboratory of Cardiovascular Molecular Biology and Regulatory Peptides Key Laboratory of Molecular Cardiovascular Science, Ministry of Education Beijing Key Laboratory of Cardiovascular Receptors Research. Peking University Third Hospital Beijing 100191 China

**Keywords:** hyperuricaemia, LncRNA‐HOTAIR, miR‐22, NLRP3 inflammasome, pyroptosis

## Abstract

Long non‐coding RNA (lncRNA) plays an important role in the renal inflammatory response caused by hyperuricaemia. However, the underlying molecular mechanisms through which lncRNA is involved in endothelial injury induced by hyperuricaemia remain unclear. In this study, we investigated the regulatory role of lncRNA‐HOTAIR in high concentration of uric acid (HUA)–induced renal injury. We established hyperuricaemia mouse model and an in vitro uric acid (UA)–induced human umbilical vein endothelial cell (HUVEC) injury model. In HUA‐treated HUVECs and hyperuricaemia mice, we observed increased HOTAIR and decreased miR‐22 expression. The expression of pyroptosis‐associated protein (NLRP3, Caspase‐1, GSDMD‐N, GSDMD‐FL) was increased. The release of LDH, IL‐1β and IL‐18 in cell supernatants and the sera of model mice was also increased. The proliferation of HUVECs stimulated by HUA was significantly inhibited, and the number of TUNEL‐positive cells in hyperuricaemia mouse kidney was increased. Bioinformatics analysis and luciferase reporter and RIP assays confirmed that HOTAIR promoted NLRP3 inflammasome activation by competitively binding miR‐22. In gain‐ or loss‐of‐function experiments, we found that HOTAIR and NLRP3 overexpression or miR‐22 knock down activated the NLRP3 inflammasome and promoted pyroptosis in HUA‐treated HUVECs, while NLRP3 and HOTAIR knockdown or a miR‐22 mimic exerted the opposite effects. Furthermore, in vivo experiments validated that HOTAIR knockdown alleviated renal inflammation in hyperuricaemia mice. In conclusion, we demonstrated that in hyperuricaemia, lncRNA‐HOTAIR promotes endothelial cell pyroptosis by competitively binding miR‐22 to regulate NLRP3 expression.

## 1 INTRODUCTION

Hyperuricaemia is a disease caused by purine metabolism disorders. It has become a risk factor for cardiovascular disease and chronic kidney disease, in addition to hypertension, diabetes, obesity, hyperlipidaemia, etc.^1^, ^2^, ^3^ Current studies have confirmed that high UA levels can cause microvascular disorders in the kidney, vascular endothelial dysfunction and the release of inflammatory cytokines,^4^, ^5^, ^6^ subsequently causing kidney damage. Renal biopsies from patients with hyperuricaemia nephropathy showed interstitial infiltration of inflammatory cells and elevated serum levels of inflammatory markers, indicating that hyperuricaemia can induce an inflammatory response and cause kidney injury.^7^, ^8^

Inflammatory damage in the kidney is inseparable from the activation of inflammasomes. In particular, the nucleotide‐binding domain and leucine‐rich repeat (NLR) protein 3 (NLRP3) inflammasome has been shown to cause acute and chronic kidney diseases by regulating canonical and non‐canonical mechanisms of inflammation.^9^, ^10^, ^11^ Therefore, the involvement of NLRP3 in kidney damage caused by high serum UA stimulation cannot be ignored in clinical practice. The NLRP3 inflammasome is composed of NLRP3, apoptosis‐associated speck‐like protein containing a CARD (ASC) and active caspase‐1, which regulates interleukin‐1β (IL‐1β) maturation.^12^, ^13^ Its activation promotes caspase‐1‐mediated cytokine maturation and simultaneously enhances the release of the N‐terminal domain of gasdermin D from the self‐inhibiting C‐terminal domain and subsequent transfer to the plasma membrane, forming large nanopores in the plasma membrane, thereby causing pyroptosis.^14^, ^15^, ^16^, ^17^ This process is accompanied by the release of a large number of pro‐inflammatory cytokines, further aggravating tissue inflammatory injury. Studies have shown that pyroptosis is associated with the occurrence and development of kidney diseases.^18^, ^19^, ^20^, ^21^ The major kidney damage caused by hyperuricaemia is endothelial cell dysfunction. However, the molecular mechanism by which HUA triggers the activation of inflammasomes and thereby promotes pyroptosis has not been elucidated.

Long non‐coding RNA (lncRNA) is a class of non‐coding RNA molecules over 200 nt in length. lncRNA plays important roles in cell differentiation, proliferation, apoptosis and pyroptosis.^22^, ^23^, ^24^ Its mechanism relies on binding with microRNAs (miRNAs) to prevent the latter from binding to target genes, thereby regulating gene expression.^25^, ^26^ Similarly, lncRNAs play important roles in the occurrence and development of kidney diseases. For example, Gm4419 increases the inflammation‐mediated inflammatory response in diabetic nephropathy through the nuclear factor‐kappa B (NF‐κB)/NLRP3 pathway.^27^ Metastasis‐associated lung adenocarcinoma transcript 1 (MALAT1) regulates high glucose–induced inflammatory responses in endothelial cells.^28^ Our previous study also confirmed that HOX transcript antisense RNA (HOTAIR) promotes the proliferation and migration of clear cell renal cell carcinoma (ccRCC) by increasing hypoxia‐inducible factor (HIF)‐1α expression via competing with miR‐217.^29^ lncRNAs can also promote the angiogenesis function of endothelial cells.^30^ These results suggest that HOTAIR might be involved in vascular endothelial cell injury induced by HUA. In acute renal injury caused by sepsis, HOTAIR promotes the apoptosis of HK‐2 cells through the miR‐22/HMGB1 pathway, thereby causing kidney injury.^31^ However, whether the interaction between HOTAIR and miR‐22 participates in vascular endothelial injury caused by HUA levels has not been reported.

Therefore, in this study, by culturing vascular endothelial cells and constructing a hyperuricaemia animal model, we attempted to confirm the molecular mechanism underlying kidney injury; that is, in an HUA environment, HOTAIR induces the activation of the NLRP3 inflammasome and pyroptosis by binding to miR‐22, ultimately promoting endothelial cell injury and causing kidney injury.

## 2 MATERIALS AND METHODS

### 2.1 Patients and controls

Seventeen hyperuricaemia patients at the Chinese PLA General Hospital were screened by the following criteria. The inclusion criteria for 17 hyperuricaemia patients (male 12 and female 5) were as follows: subjects are between the age of 21 and 85, with an average age of 43.5 ± 3.7 years; subjects meet the diagnostic criteria of hyperuricaemia (serum UA concentration >440 μmol/L for males and >367 μmol/L for females); first onset; informed consent. The exclusion criteria were as follows: patients with stage 3 hypertension, diabetic nephropathy and anti‐UA therapy in the previous 3 months. Among those included, 12 were male, and five were female, with an average age of 43.5 years. Additionally, we selected 17 age‐matched healthy volunteers as controls. Fasting blood samples were collected from all subjects and centrifuged at 3000 g for 10 min at 4°C. Serum samples were then collected for detecting the expression levels of HOTAIR, miR‐22, IL‐1β and IL‐18 using qPCR analysis and ELISA. This study was approved by the Ethics Committee of PLA General Hospital, and informed consent was obtained from all participants.

### 2.2 Cell culture

Human umbilical vein endothelial cells (HUVECs) (American Type Culture Collection, No. CRL‐1730) were cultured in endothelial culture medium (ECM) (ScienCell Research Laboratories, cat. no. 1001) containing 5% FBS, 1% endothelial cell growth supplement and 1% penicillin/streptomycin solution in a 37°C, 5% CO_2_ incubator. When cell confluence reached 50%, 600 μmol/L UA (Sigma‐Aldrich, cat. no. U2625) was added and then incubated for 24 h.

### 2.3 Lentivirus construction and cell transfection

To overexpress lncRNA‐HOTAIR or NLRP3, the full‐length cDNA fragment of human lncRNA‐HOTAIR or NLRP3 was cloned into the lentiviral vector pReceiver‐Lv185 (FulenGen), and 293T cells were used to produce pReceiver‐Lv185‐HOTAIR and pRevceiver‐NLRP3. In addition, lentivirus vectors psiH1‐HOTAIR and psiH1‐NLRP3 were used to knock down the above 2 genes. A miR‐22 mimic and inhibitor (AMO‐22) were designed and manufactured by Sangon Biotech. HUVECs were infected with the lentiviral vectors when they reached 70%–80% confluence.

### 2.4 Dual‐luciferase reporter assay

Using GenBank as a reference, the fragment of the NLRP3 (NM004895) 3′‐untranslated region (UTR) containing the miR‐22 targeting sequence (5′‐AAGCGU‐3′) was cloned, via PCR, into the psiCHECKTM‐2 dual‐luciferase reporter plasmid (Promega, cat. no. E1910) to produce psiCHECK‐WT‐NLRP3 and the mutant vector psiCHECK‐MT‐NLRP3. Then, the two plasmids were co‐transfected into HUVECs with either miR‐22 mimic, inhibitor or miR‐22 control. For the reporter assays, HUVECs were harvested and lysed and then the assays were conducted according to manufacturer's instructions (Promega, cat. no. E1910). Firefly and Renilla luciferase activities were measured using a dual‐luciferase reporter assay system. Firefly luciferase activity (*F* value) and Renilla luciferase activity (*R* value) were detected using a fluorescence/chemiluminescence instrument. The ratio of Renilla luciferase activity to firefly luciferase activity represented the relative expression level of reporter genes. Similarly, the region containing the target sequence in human lncRNA‐HOTAIR was cloned into the vector psiCHECKTM‐2, and similar experiments were carried out.

### 2.5 RNA‐binding protein immunoprecipitation (RIP) assay

RIP assays were performed using a Magna RIP RNA‐Binding Protein Immunoprecipitation Kit (Sigma‐Aldrich, cat. no. 17‐700). HUVECs were lysed in RIP lysis buffer, and subsequently, 100 μl of cell lysate was incubated with RIP buffer containing magnetic beads conjugated with human anti‐Ago2 antibody (1:50 dilution, Millipore) and corresponding negative normal control IgG (Millipore). The samples were incubated with proteinase K buffer, and target RNA was extracted for further study. qPCR assays were conducted to determine HOTAIR and miR‐22 levels in the precipitates.

### 2.6 RNA pulldown assay

RNA pulldown assay was carried out by using a Pierce™ Magnetic RNA‐Protein Pull‐Down Kit (Thermo Fisher, cat. no. 20164). Briefly, RNAs were biotin‐labelled and in vitro transcribed with Biotin RNA Labeling Mix and T7/SP6 RNA polymerase. Cells were lysed and incubated with biotinylated RNAs. After treated with RNase‐free DNase I (Roche, cat. no. 69182) and then purified with the RNeasy Mini Kit (Qiagen, cat. no. 74104), the bound RNAs were extracted for further evaluation by qRT‐PCR analysis.

### 2.7 Animals and treatment

Eight‐ to twelve‐week‐old C57/B6 male mice were provided by the Experimental Animal Center of the Academy of Military Medical Sciences (a total of 24 mice: six mice in HUA model group, six mice in HUA + Vector group and six mice in HUA + shHOTAIR group, six for control group); the animal were allowed to adapt for 1 week before the experiments. Hyperuricaemia was induced by gavage of potassium oxonate (PO; Sigma‐Aldrich, cat. no. U156124) (250 mg/kg·day) and UA (Sigma‐Aldrich, cat. no. U2625) solution (250 mg/kg·day) once a day for 10 consecutive days. A UA assay kit (abcam, cat. no. Ab65344) was used to detect serum UA levels in mice and determine whether modelling was successful.

### 2.8 CCK‐8 assay

HUVECs in 96‐well plates (1000–2000 cells per well) were treated with 600 µmol/L UA solution for 0 day, 1 day, 2 days, 3 days, 4 days, 5 days, 6 days or 7 days, and then, the culture medium was removed. The cells were washed twice with phosphate‐buffered saline (PBS, Beyotime, cat. no. C0221A), and then, 10 µl of CCK‐8 solution (Dojindo, cat. no. CK04) was added to each well. The cells were incubated at 37°C for 2 h, and then, the absorbance (450 nm) for each well was determined using an ELISA reader (Bio‐Rad, iMARK).

### 2.9 LDH release assay

To test cell cytotoxicity, we used a lactate dehydrogenase assay kit (abcam, cat. no. ab102526) to detect LDH activity in serum and cell culture supernatant. Samples were placed into a 96‐well plate, and 50 µl of reaction mixture (LDH assay buffer, 48 µl; LDH substrate mix, 2 µl) was added. After incubating 45 min at 37°C, the absorbance (450 nm) for each well was determined using an ELISA reader (Bio‐Rad, iMARK).

### 2.10 RNA extraction and qPCR

Total RNA was extracted from patient serum, mouse kidney tissue and HUVECs using TRIzol reagent (Invitrogen, cat. no. 15596026) following the manufacturer's protocol. One microgram of total RNA was reverse transcribed into cDNA using a First Strand cDNA Synthesis Kit (TOYOBO, cat. no. FSK‐101) according to the manufacturer's instructions. The qPCR system was prepared with a SYBR Select Master Mix kit (Thermo Fisher, cat. no. 4472903), and then, the relative expression levels of HOTAIR and miR‐22 were determined using a 7500 FAST real‐time PCR system (Applied Biosystems) according to standard procedures. 18s rRNA was used for normalization. The primers are provided in Table 1.

**TABLE 1:** qPCR primer sequences

	Forward	Reverse
HOTAIR (murine)	5′‐CCTTATAAGCTCATCGGAGCA‐3′	5′‐CATTTCTGGGTGGTTCCTTT‐3′
HOTAIR (human)	5′‐CAGTGGGGAACTCTGACTCG‐3′	3′‐GTGCCTGGTGCTCTCTTACC‐3′
miR‐22 (murine)	5′‐ACACTCCAGCTGGGTTCGACGGTCAACTTC‐3′	5′‐CTCAACTGGTGTCGTGGAGTCGGCAATTCAGTTGAGACAGTTCT‐3′
miR‐22 (human)	5′‐GGTTAAGCTGCCAGTTGAA‐3′	5′‐CCAGTGCGTGTCGTGGAGT‐3′

### 2.11 Western blot

Total protein was extracted from mouse kidney tissue and HUVECs using RIPA lysis buffer (Beyotime, cat. no. P0013B) on ice; the obtained lysate was centrifuged at 12,000 × g at 4°C for 30 min. Protein quantification was performed by using a BCA Protein Assay Kit (Thermo Fisher, cat.no. A53225). Proteins were separated by 10% SDS‐PAGE and then transferred to an NC membrane (Pall Bio Trace, cat. no. 66485) using a Bio‐Rad Trans‐Blot Turbo (USA) system. After blocking with 5% fat‐free milk powder in TBST buffer, the membranes were incubated with the following primary antibodies overnight at 4°C: anti‐GAPDH (abcam, cat. no. ab9485), anti‐caspase‐1 (Cell Signaling Technology, cat. no. 3866), anti‐NLRP3 (Cell Signaling Technology, cat. no. 13158), anti‐NLRP1 (Cell Signaling Technology, cat. no. 4990), anti‐GSDMD‐FL (Invitrogen, cat. no. PA5‐104324) and anti‐GSDMD‐N (abcam, cat. no. ab215203). Next, the membranes were incubated with horseradish peroxidase–conjugated anti‐rabbit IgG secondary antibodies for 2 h at room temperature. Bands were detected using a ChemiDoc MP Imaging System (Bio‐Rad), and ImageJ 1.51 software was used for the quantitative analysis of all protein bands.

### 2.12 Terminal deoxynucleotidyl transferase dUTP nick end labelling (TUNEL) staining

Paraffin sections of mouse kidney tissue were dewaxed in water. TUNEL staining was performed according to the kit instructions. The sections were stained with 3, 3′‐diaminobenzidine (DAB; Servicebio, cat. no. G2111), and the nuclei were stained with haematoxylin. TUNEL‐positive cells, which were stained brown, were counted under 400× magnification. The pyroptosis rate was calculated as the percentage of TUNEL‐positive cells relative to the total number of renal tubular cells. Nuclei stained with haematoxylin were blue, and pyroptosis nuclei stained with DAB were brownish‐yellow.

### 2.13 Immunofluorescent staining

Frozen sections of mouse kidney tissue were permeabilized in 0.2% Triton X‐100 for 15 min and blocked with 5% bovine serum albumin (BSA; Amresco, cat. no. 0332‐250G) at room temperature for 1 h. Subsequently, the sections were incubated with following antibodies overnight at 4°C: anti‐caspase‐1 (Cell Signaling Technology, Cat. No. 3866), NLRP3 (Cell Signaling Technology, cat. no. 13158) or GSDMD‐FL (Invitrogen, cat. no. Pa5‐104324). Then, the sections were incubated with Cy3‐conjugated secondary anti‐rabbit IgG antibody (Beyotime, cat. no. A0516) at room temperature in the dark for 1 h. The sections were incubated with DAPI (abcam, cat. no. ab104139) for 15 min. The sections were washed with PBS three times after each incubation. Fluorescence was assessed with a laser scanning confocal microscope (Leica).

### 2.14 Immunohistochemical staining

Renal samples were fixed in 4% paraformaldehyde and embedded in paraffin. The samples were then cut into 5‐µm‐thick sections and incubated with primary antibody, that is anti‐CD68 (abcam, cat. no. ab955), at 4°C overnight, followed by incubation with the appropriate secondary antibody. Then, the sections were stained with diaminobenzidine, and images were captured using a fluorescence microscope (Olympus).

### 2.15 ELISA

Twenty‐four hours after transfection, the concentrations of IL‐1β (abcam, cat. no. ab100562) and IL‐18 (Novus, cat. no. KA0561) in HUVEC culture supernatants or HUA mouse sera were determined by ELISA. The detection was performed strictly according to the kit instructions.

### 2.16 Statistical analysis

Statistical analysis was performed using IBM SPSS Statistics 24.0 software (IBM Corporation). The quantitative data are expressed as the mean ± standard deviation (SD). All data were tested for a normal distribution using the Shapiro‐Wilk test. Comparisons between two groups were performed with Student's *t* test, and multiple comparisons of more than two groups were performed with one‐way analysis of variance (ANOVA). A *p* value less than 0.05 was considered to be statistically significant.

## 3 RESULTS

### 3.1 High UA levels promote an increase in HOTAIR expression and induce inflammation

**FIGURE 1:**
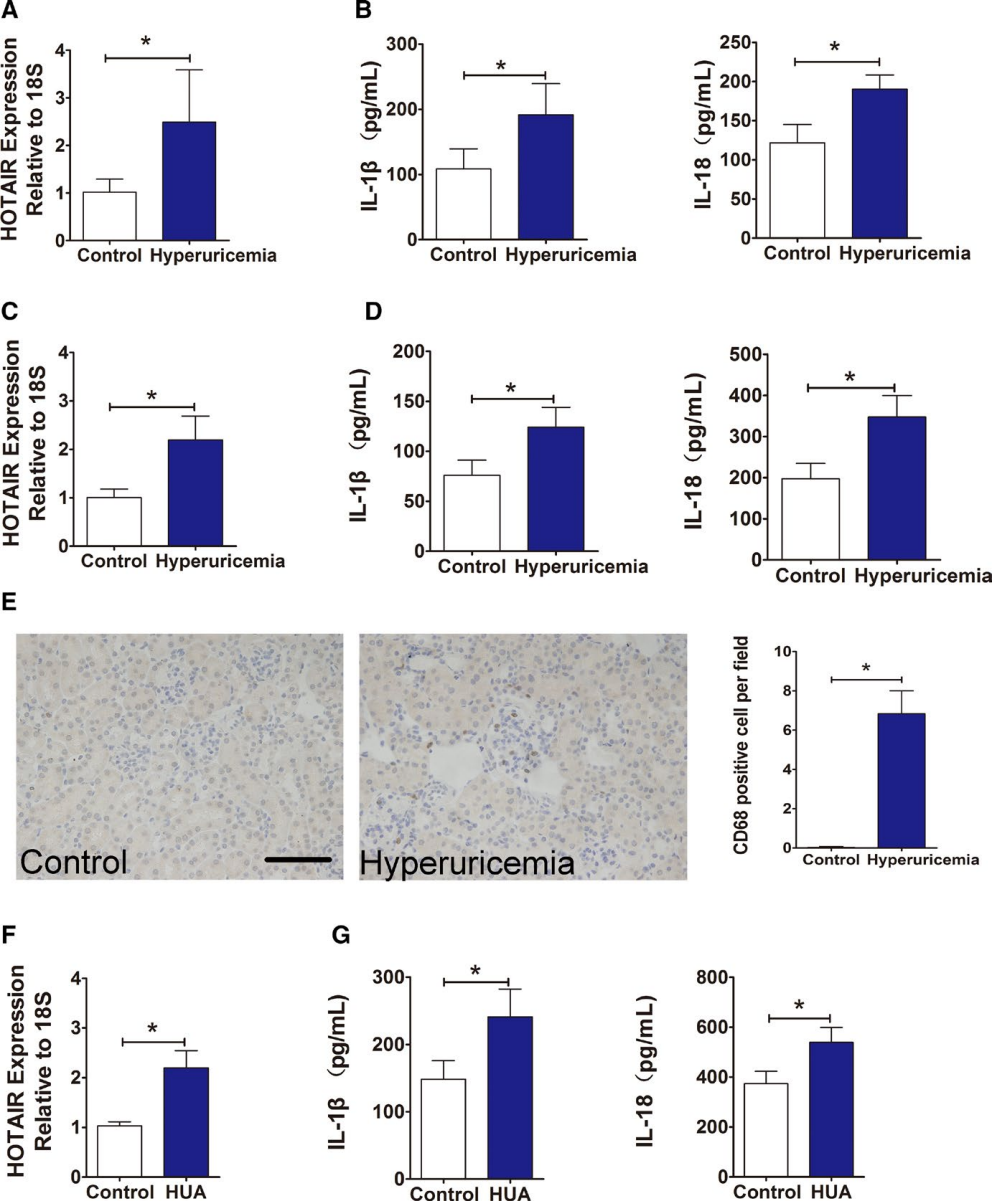
Effect of HUA on inflammation and the expression of lncRNA‐HOTAIR in patients, hyperuricaemic mice and HUVECs. (A) Serum levels of HOTAIR in normal controls and hyperuricaemia patients, measured by qPCR. HOTAIR expression was significantly increased in hyperuricaemia group; ∗*p* < 0.05 compared to the normal controls; *n* = 17 in each group. (B) Serum levels of IL‐1β and IL‐18 in normal controls and hyperuricaemia patients, measured by ELISA. Serum IL‐1β and IL‐18 levels were significantly higher in hyperuricaemia group; ∗*p* < 0.05 compared to the normal controls; *n* = 17 in each group. (C) Serum levels of HOTAIR in control mice and hyperuricaemia mice, measured by qPCR. HOTAIR expression was significantly higher in the hyperuricaemia mice; ∗*p* < 0.05 compared to the control group; *n* = 6 in each group. (D) Serum levels of IL‐1β and IL‐18 in control mice and hyperuricaemia mice, measured by and ELISA. IL‐1β and IL‐18 levels were significantly higher in hyperuricaemia group; ∗*p* < 0.05 compared to the control group; *n* = 6 in each group. (E) Immunohistochemistry of CD68 in renal tissue from hyperuricaemia mice and control mice. Positive CD68 staining implies the infiltration of mononuclear macrophages in the renal interstitium and glomerular mesangial area in hyperuricaemia group; ∗*p* < 0.05 compared to the control group; *n* = 6 in each group (scale bar, 100 μm; magnification, 400×). (F) HOTAIR levels in HUA‐treated HUVECs, measured by qPCR. HOTAIR expression was significantly higher in the HUA group; ∗*p* < 0.05 compared to the control group; *n* = 3 in each group. (G) IL‐1β and IL‐18 levels in HUA‐treated HUVECs, measured by ELISA. IL‐1β and IL‐18 levels were significantly higher in HUA group; ∗*p* < 0.05 compared to the control group; *n* = 3 in each group

Serum from patients with hyperuricaemia and healthy individuals was collected for qPCR analysis. Compared with that in serum from healthy individuals, the expression of HOTAIR in serum from hyperuricaemia patients was significantly higher (Figure 1A). Serum levels of inflammatory cytokines were determined by ELISA. The results showed that compared to those in the healthy control group, the serum inflammatory cytokine levels of IL‐1β and IL‐18 in hyperuricaemia patients were significantly higher (Figure 1B). Similarly, in the hyperuricaemia mouse model, the expression of HOTAIR, as determined by qPCR, in the kidney tissue of model mice was significantly higher than that in tissue from the healthy control group (Figure 1C), and the serum inflammatory cytokines IL‐1β and IL‐18 were also significantly higher in the model mice than in the control group (Figure 1D). Furthermore, immunohistochemical staining of the macrophage marker CD68 in renal tissue sections showed that CD68+ macrophage infiltration increased around blood vessels in the glomerular mesangial area and renal interstitium in hyperuricaemia mice; statistical analysis indicated that the difference between the groups was significant (Figure 1E).

Subsequently, HUVECs were cultured and stimulated with 600 μmol/L UA for 24 h. qPCR showed that the expression of HOTAIR in cells stimulated with HUA group was significantly higher than that in cells of the control group (Figure 1F); furthermore, the levels of the inflammatory cytokines IL‐1β and IL‐18 in the culture supernatant was also significantly higher in the HUA group (Figure 1G).

### 3.2 HUA levels induce NLRP3 inflammasome activation and pyroptosis in endothelial cells

**FIGURE 2:**
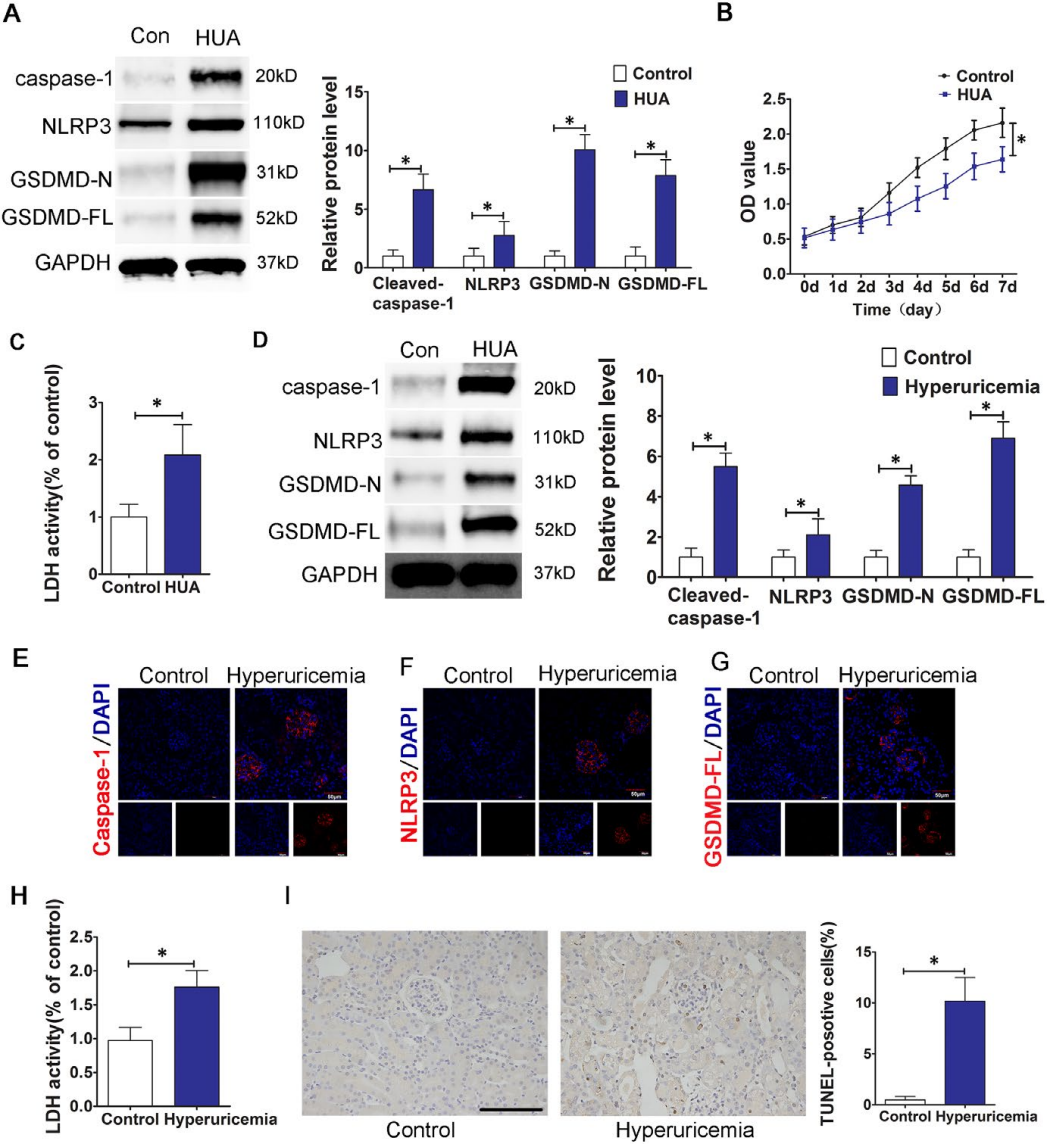
HUA induces the activation of the NLRP3 inflammasome and pyroptosis. (A) The protein levels of caspase‐1, NLRP3, GSDMD‐N and GSDMD‐FL, as measured by Western blot analysis; quantification normalized to GAPDH in HUA‐treated HUVECs. The HUA group showed increased caspase‐1, NLRP3, GSDMD‐N, GSDMD‐FL protein expression levels after 24 h of stimulation; *p* < 0.05 compared to the control group; *n* = 3 in each group. (B) Cell viability of HUA‐treated HUVECs was determined by the CCK‐8 assay. The growth of HUVECs was significantly inhibited in HUA group. *p < 0.05 compared to the control group; *n* = 3 in each group. (C) LDH secretion in HUA‐treated cells was assessed using a commercial kit. LDH release increased significantly in HUA group. *p < 0.05 compared to the control group; *n* = 3 in each group. (D) The protein levels of caspase‐1, NLRP3, GSDMD‐N and GSDMD‐FL, as measured by Western blot analysis; quantification normalized to GAPDH in renal tissue from hyperuricaemia mice. The protein levels of caspase‐1, NLRP3, GSDMD‐N and GSDMD‐FL increased significantly in hyperuricaemia group. *p < 0.05 compared to the control group; *n* = 6 in each group. (E–G) Immunofluorescence images showing the expression of caspase‐1, NLRP3 and GSDMD‐FL in renal tissue from hyperuricaemia mice. Tissue immunofluorescence showed that the fluorescence intensity of glomerular NLRP3, caspase‐1 and GSDMD‐FL in the hyperuricaemia group was significantly higher than that in the control group. *p < 0.05 compared to the control group; *n* = 6 in each group (scale bar, 50 μm; magnification, 400×); blue: nuclear staining (DAPI), red: caspase‐1, NLRP3 and GSDMD‐FL staining. (H) Levels of LDH in mouse serum, measured using a commercial kit. LDH release increased significantly in the hyperuricaemia group. *p < 0.05 compared to the control group; *n* = 6 in each group. (I) TUNEL staining of renal tissue sections from control mice or hyperuricaemia mice. The number of positive cells in the kidney of HUA mice increased significantly in hyperuricaemia group. *p < 0.05 compared to the control group; *n* = 6 in each group (scale bar, 100 μm; magnification, 400×); blue, nuclear staining (DAPI)

To determine whether high UA levels activate the NLRP3 inflammasome in endothelial cells and to assess the effect on pyroptosis, HUVECs were stimulated with 600 μmol/L UA for 24 h. Western blot results showed that compared with that in the control group, the expression of NLRP3 increased and the expression of pyroptosis‐related proteins, including caspase‐1, GSDMD‐FL and GSDMD‐N, significantly increased in the HUA treatment group (Figure 2A). The CCK‐8 method was used to examine the effect of HUA on the proliferation of HUVECs. The results showed that compared with that of cells in the control group, the growth of the cells in the experimental group was significantly inhibited (Figure 2B). Furthermore, LDH in the culture supernatant was analysed using an LDH assay kit; LDH release in the experimental group was also significantly higher (Figure 2C).

Furthermore, we examined the status of inflammasome activation and pyroptosis in kidney tissue from hyperuricaemia mice. The results showed that the protein expression levels of NLRP3, caspase‐1, GSDMD‐FL and GSDMD‐N in kidney tissue from hyperuricaemia mice were significantly higher than those in kidney tissue from mice in the saline control group (Figure 2D). Tissue immunofluorescence results indicated that the expression of NLRP3, caspase‐1 and GSDMD‐FL was distributed throughout the glomeruli of the hyperuricaemia group; the glomerular fluorescence intensity in the hyperuricaemia group was significantly higher than that in the control group (Figure 2E–G). Similarly, compared with that in the control group, serum LDH in hyperuricaemia mice was significantly higher (Figure 2H), and the number of kidney TUNEL‐positive cells was significantly higher (Figure 2I). Both in vitro and in vivo experimental results indicated that HUA induced NLRP3 inflammasome activation and pyroptosis in the cells.

### 3.3 miR‐22 binds to HOTAIR and NLRP3

**FIGURE 3:**
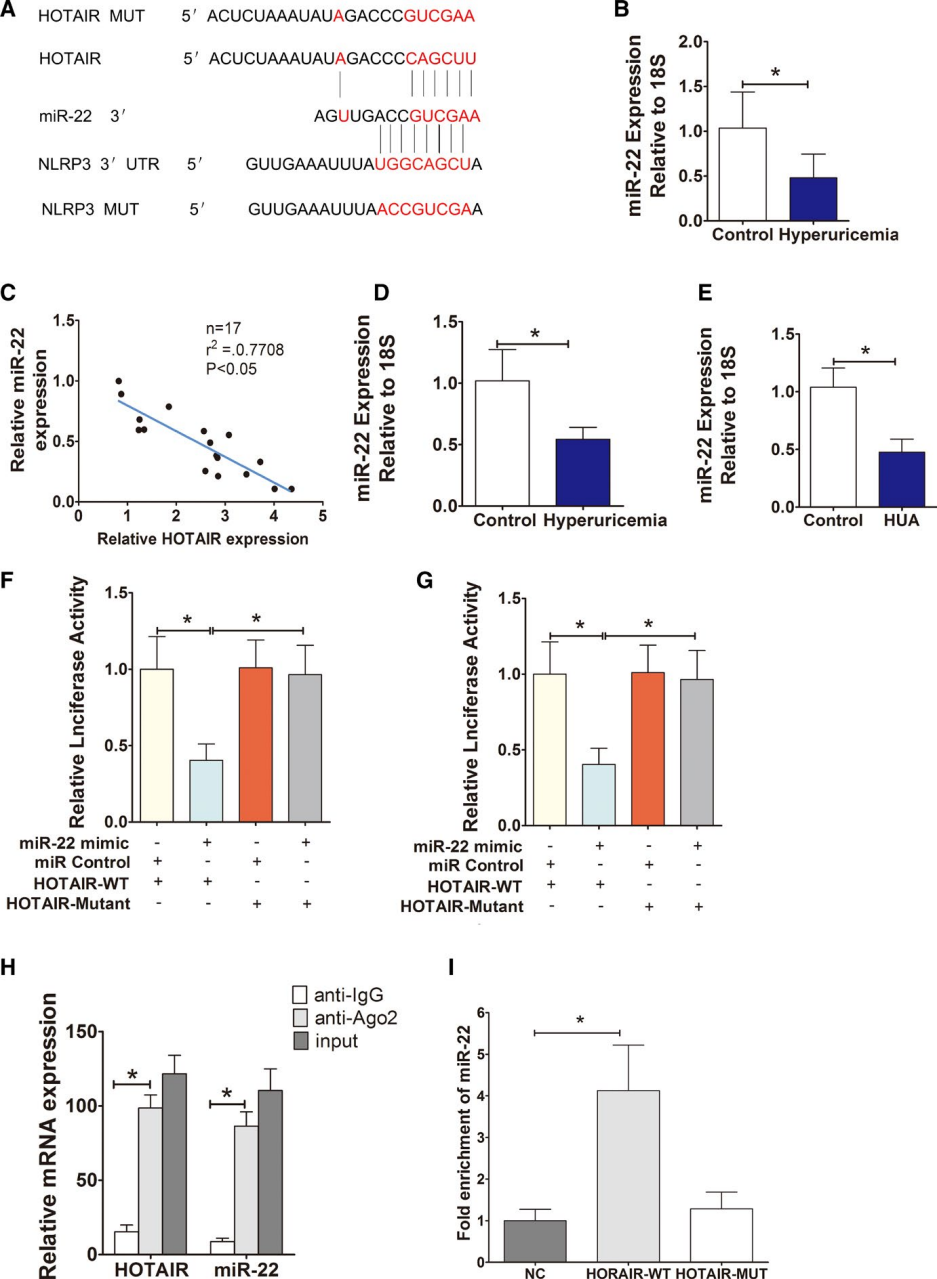
HOTAIR, as a ceRNA, regulates the expression of miR‐22. (A) The sequences of HOTAIR and NLRP3 aligned with miR‐22, including the wild type (WT) and a mutant. Schematic illustration of the presumed target site for HOTAIR and NLRP3 in miR‐22. (B) Serum levels of miR‐22 in normal controls and hyperuricaemia patients, measured by qPCR. The expression of miR‐22 in hyperuricaemia group was significantly down‐regulated and negatively correlated with the expression of HOTAIR; ∗*p* < 0.05 compared to the control group; *n* = 17 in each group. (C) Correlation analysis between HOTAIR and miR‐22 levels in normal controls and hyperuricaemia patients. miR‐22 was negatively correlated with HOTAIR (*r* ^2^ = −0.77); ∗*p* < 0.05 compared to the normal controls; *n* = 17 in each group. (D,E) The levels of miR‐22 in hyperuricaemia mice (*n* = 6) and HUVECs (*n* = 3), measured by qPCR. The expression of miR‐22 in HUA mice and HUA‐stimulated HUVECs was significantly down‐regulated; ∗*p* < 0.05 compared to the control group; *n* = 6 in each group. (F) Luciferase activity results. There is direct binding between HOTAIR and miR‐22; ∗*p* < 0.05 vs. the mimic NC+HOTAIR‐WT group; *n* = 3 in each group. (G) Luciferase activity results. miR‐22 directly regulates the expression of NLRP3, ∗*p* < 0.05 compared to the mimic NC+NLRP3‐WT group; *n* = 3 in each group. (H) RIP assays using cell lysate IgG or anti‐Ago2 as the input. Relative expression levels of HOTAIR and miR‐22 in HUVECs were detected by qPCR and normalized to 18s. The results indicated higher HOTAIR and miR‐22 RNA levels in Ago2 immunoprecipitates relative to control IgG immunoprecipitates; ∗*p* < 0.05 compared to the anti‐IgG group; *n* = 3 in each group. (I) Detections of miR‐22 using qRT‐PCR in the same sample pulled down by biotinylated HOTAIR and NC probe. miR‐22 expression was significantly higher in the HOTAIR‐WT group; ∗*p* < 0.05 compared to NC group; *n* = 3 in each group

To explore the regulatory relationship between HOTAIR and NLRP3, we used bioinformatics prediction and analysis (www.mircode.org), and the results suggest that HOTAIR might be a competing endogenous RNA (ceRNA) of miR‐22. HOTAIR and miR‐22 have potential binding sites (Figure 3A). qPCR detection showed that miR‐22 expression in serum from HUA patients was significantly down‐regulated; there was a strong negative correlation between miR‐22 and HOTAIR expression (*r* ^2^ = −0.77) (Figure 3B,C). Similarly, miR‐22 expression was significantly down‐regulated in serum from HUA mice (Figure 3D). miR‐22 down‐regulation was also observed in endothelial cells cultured in UA (Figure 3E). Furthermore, the mutated sequence of full‐length HOTAIR or the seed region was cloned into the psiCHECK2 vector and co‐transfected into HEK‐293T cells with a miR‐22 mimic or a miR‐22 inhibitor. The luciferase assay results showed that the miR‐22 mimic significantly inhibited the luciferase activity of psiCHECK‐WT‐HOTAIR; however, the mimic did not significantly inhibit the luciferase activity of mutant MT‐HOTAIR (Figure 3F).

TargetScan (www.targetscan.org) online software analysis suggested that there was a miR‐22 binding region in the 3‐UTR of NLRP3, suggesting that NLRP3 may be the target gene of miR‐22 and NLRP3 and miR‐22 have potential binding sites (Figure 3A). Subsequently, a luciferase reporter assay confirmed that the miR‐22 mimic significantly inhibited luciferase activity in wild‐type psiCHECK‐WT‐NLRP3 but had no significant inhibitory effect on the luciferase activity of psiCHECK‐MT‐NLRP3 (Figure 3G).

miRNAs form an RNA‐induced silencing complex (RISC) with RNA via Ago2 and then induce gene silencing.^32^ Based on the above results, we speculated that HOTAIR might bind with miR‐22 and Ago2 to form a RISC, which was further verified by the RIP experiment. The results showed that the amounts of HOTAIR and miR‐22 that immunoprecipitated with Ago2 were significantly higher than those that precipitated with the IgG control (Figure 3H).

Finally, we applied a biotin‐labelled pulldown assay to further explore whether miR‐22 and HOTAIR could direct binding. Consistent with luciferase results, miR‐22 was precipitated by wild‐type HOTAIR but not HOTAIR mutant (Figure 3I).

### 3.4 HOTAIR acts as a molecular sponge to adsorb miR‐22 and cause endothelial cell inflammation and pyroptosis

**FIGURE 4:**
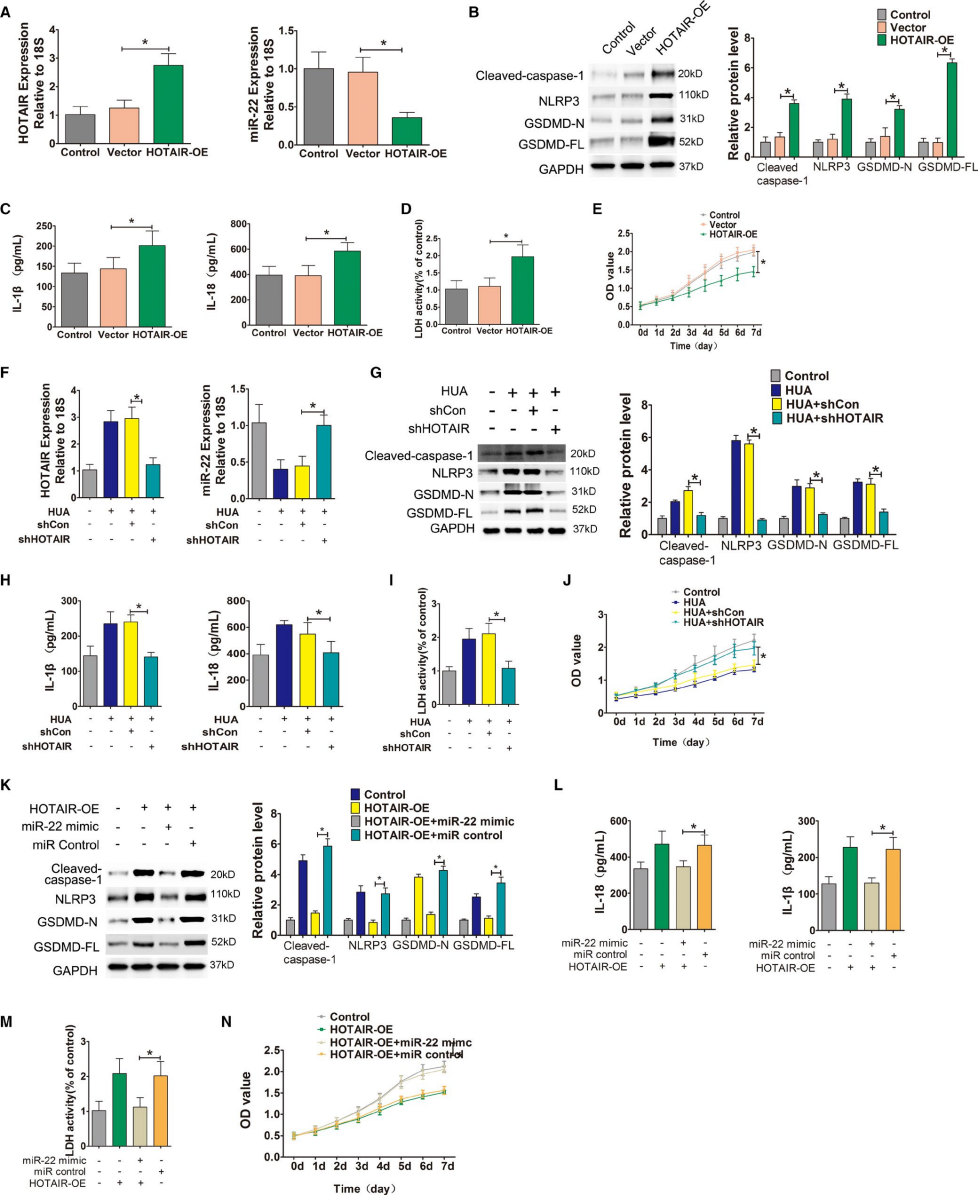
HOTAIR, as a molecular sponge, down‐regulates miR‐22. (A) Relative HOTAIR and miR‐22 expression in HUVECs, measured by qPCR after transfection. HOTAIR was significantly upregulated and miR‐22 was significantly down‐regulated in the HOTAIR‐OE group; *p < 0.05 compared to the vector group; *n* = 3 in each group. (B) Protein levels of caspase‐1, NLRP3, GSDMD‐N and GSDMD‐FL, were measured by Western blot analysis; quantification normalized to GAPDH in HUVECs that were transfected with lentiviruses. After transfection, NLRP3, caspase‐1, GSDMD‐N and GSDMD‐FL protein expression levels increased in the HOTAIR‐OE group; *p < 0.05 compared to the vector group; *n* = 3 in each group; OE: overexpression. (C) Levels of IL‐1β and IL‐18 in cell culture supernatants were measured by ELISA after transfection. After transfection, the levels of IL‐1β and IL‐18 in the culture supernatant significantly increased in HOTAIR‐OE group; *p < 0.05 compared to the vector group; *n* = 3 in each group. (D) LDH secretion was assessed using a commercial kit. After infection, LDH release increased significantly in the HOTAIR‐OE group; *p < 0.05 compared to the vector group; *n* = 3 in each group. (E) Cell viability was determined by the CCK‐8 assay after transfection. The growth of HUVECs in the HOTAIR group was significantly inhibited. *p < 0.05 compared to the vector group; *n* = 3 in each group. (F) Relative HOTAIR and miR‐22 expression in HUA‐treated cells, measured by qPCR after shRNA transfection. miR‐22 was significantly upregulated and HOTAIR was significantly down‐regulated in HUA+shHOTAIR group; *p < 0.05 compared to the HUA+shCon group; *n* = 3 in each group. (G) Protein levels of caspase‐1, NLRP3, GSDMD‐N and GSDMD‐FL, as measured by Western blot analysis; quantification normalized to GAPDH in HUVECs that were transfected with shRNA before HUA stimulation. The protein expression levels of NLRP3, caspase‐1, GSDMD‐N and GSDMD‐FL decreased in HUA+shHOTAIR group; *p < 0.05 compared to the HUA+shCon group; *n* = 3 in each group. (H) Levels of IL‐1β and IL‐18 in cell culture supernatants, measured by ELISA. The levels of IL‐1β and IL‐18 in culture supernatants significantly decreased in HUA+shHOTAIR group; *p < 0.05 compared to the UA+shCon group; *n* = 3 in each group. (I) LDH secretion was assessed using a commercial kit. LDH release decreased significantly in HUA+shHOTAIR group; *p < 0.05 compared to the HUA+shCon group; *n* = 3 in each group. (J) Cell viability was determined by the CCK‐8 assay after shRNA transfection. The proliferation of HUVECs in HUA+shHOTAIR group significantly increased; *p < 0.05 compared to the HUA+shCon group; *n* = 3 in each group. (K) Protein levels of caspase‐1, NLRP3, GSDMD‐N and GSDMD‐FL, as measured by Western blot analysis; quantification normalized to GAPDH after adding a miR‐22 mimic. The miR‐22 mimic +HOTAIR‐OE group showed decreased NLRP3, caspase‐1, GSDMD‐N and GSDMD‐FL protein expression levels after incubation with the miR‐22 mimic; *p < 0.05 compared to the HOTAIR‐OE+miR control group; *n* = 3 in each group. (L) Levels of IL‐1β and IL‐18 in cell culture supernatants, measured by ELISA. The levels of IL‐1β and IL‐18 in the miR‐22 mimic +HOTAIR‐OE group significantly decreased; *p < 0.05 compared to the HOTAIR‐OE+miR control group; *n* = 3 in each group. (M) LDH secretion was assessed using a commercial kit. LDH release decreased significantly in the miR‐22 mimic +HOTAIR‐OE group; *p < 0.05 compared to the HOTAIR‐OE+miR control group; *n* = 3 in each group. (N) Cell viability was determined using the CCK‐8 assay after adding the miR‐22 mimic. The proliferation of HUVECs in the miR‐22 mimic +HOTAIR‐OE group significantly increased. *p < 0.05 compared to the HOTAIR‐OE+miR control group; *n* = 3 in each group

We constructed and obtained a HOTAIR overexpression lentivirus, which was used to infect HUVECs that were stimulated and cultured in UA. The qPCR results showed that compared with the no‐load vector group, the HOTAIR‐transfected group showed significantly upregulated HOTAIR expression and significantly down‐regulated miR‐22 (Figure 4A). Western blot results showed that after HOTAIR overexpression in endothelial cells, the expression levels of the NLRP3 inflammasome, caspase‐1 and the pyroptosis‐related proteins GSDMD‐N and GSDMD‐FL were all upregulated (Figure 4B). In addition, the levels of the inflammatory cytokines IL‐1β and IL‐18 in the supernatant of cells overexpressing HOTAIR significantly increased. The release of LDH also significantly increased. Cell proliferation was significantly lower in the HOTAIR‐transfected group than in the no‐load vector group (Figure 4C–E).

However, after using a lentivirus to overexpress shRNA and knock down HOTAIR in HUVECs stimulated with HUA, the opposite effects were observed. Compared with the unrelated shRNA vector group (scramble), in the knockdown group, the expression of HOTAIR was down‐regulated and miR‐22 was significantly upregulated in HUVECS (Figure 4F). The expression levels of the NLRP3 inflammasome, caspase‐1 and pyroptosis‐related proteins GSDMD‐N and GSDMD‐FL were all down‐regulated (Figure 4G), the release of the inflammatory cytokines IL‐1β, IL‐18 and LDH was reduced (Figure 4H,I), and cell proliferation significantly increased (Figure 4J).

On this basis, cells overexpressing HOTAIR were co‐transfected with a miR‐22 mimic. Compared to that in the microRNA scramble control group, the expression levels of the NLRP3 inflammasome, caspase‐1, and pyroptosis‐related proteins GSDMD‐N and GSDMD‐FL in the cells co‐transfected with the mimic all decreased (Figure 4K); in addition, the release of the inflammatory cytokines IL‐1β and IL‐18 and LDH in the culture supernatant significantly decreased (Figure 4L,M), and cell proliferation significantly increased (Figure 4N).

### 3.5 High UA promoted endothelial cell inflammation and pyroptosis via HOTAIR/miR‐22

**FIGURE 5:**
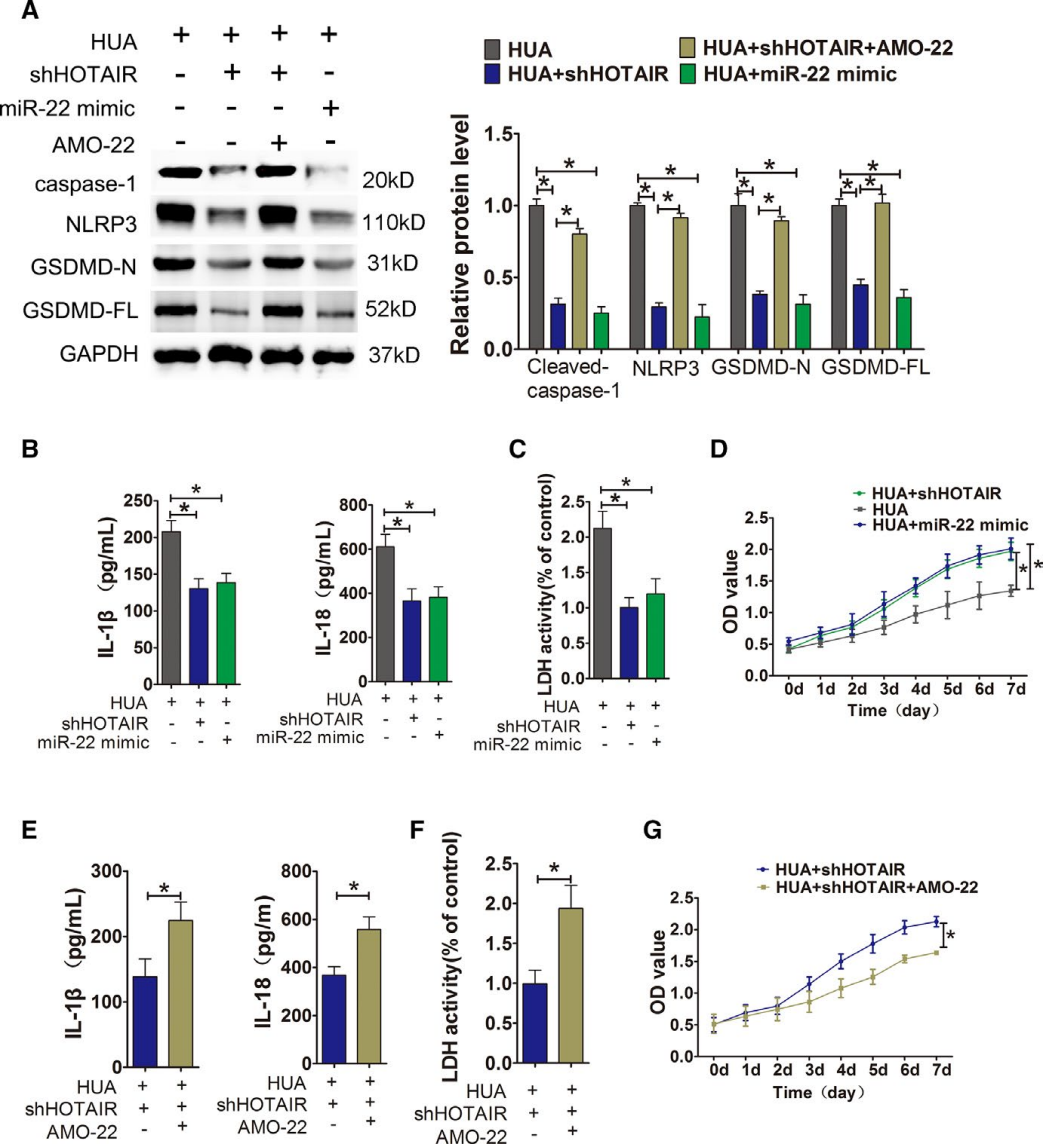
HUA promoted HUVECs inflammation and pyroptosis through HOTAIR/miR‐22. (A) Protein levels of caspase‐1, NLRP3, GSDMD‐N and GSDMD‐FL, as measured by Western blot analysis; quantification normalized to GAPDH after incubation with miR‐22 mimic or AMO‐22 after HIOTAIR knockdown. The expression of NLRP3, caspase‐1, GSDMD‐N and GSDMD‐FL decreased in UA+shHOTAIR group and UA+miR‐22 mimc group; *p < 0.05 compared to the UA group. The expression of NLRP3, caspase‐1, GSDMD‐N and GSDMD‐FL increased in UA+shHOTAIR+AMO‐22 group; *p < 0.05 compared to the UA+shHOTAIR group; *n* = 3 in each group. (B,C) Levels of IL‐1β, IL‐18 and LDH in cell culture supernatants, measured by ELISA after incubation with miR‐22 mimic after HIOTAIR knockdown. The levels of IL‐1β, IL‐18 and LDH decreased in UA+shHOTAIR group and UA+miR‐22 mimc group; *p < 0.05 compared to the UA group; *n* = 3 in each group. (D) Cell viability was determined by the CCK‐8 assay after incubation with miR‐22 mimic after HIOTAIR knockdown. The proliferation of UA+shHOTAIR group and UA+miR‐22 mimc group significantly increased; *p < 0.05 compared to the UA group; *n* = 3 in each group. (E,F) Levels of IL‐1β, IL‐18 and LDH in cell culture supernatants, measured by ELISA after incubation with AMO‐22 after HIOTAIR knockdown. The levels of IL‐1β, IL‐18 and LDH significantly increased in UA+shHOTAIR+AMO‐22 group; *p < 0.05 compared to UA+shHOTAIR group; *n* = 3 in each group. (G) Cell viability was determined by the CCK‐8 assay after incubation with AMO‐22 after HIOTAIR knockdown. The proliferation of UA+shHOTAIR+AMO‐22 group significantly decreased; *p < 0.05 compared to UA+shHOTAIR group; *n* = 3 in each group

To confirm whether HUA promoted endothelial cell inflammation and pyroptosis via HOTAIR/miR‐22, we added miR‐22 mimic and AMO‐22 to UA‐treated HUVECs after HOTAIR knockdown by lentivirus, respectively. The expression levels of the pyroptosis‐related proteins NLRP3, caspase‐1, GSDMD‐N and GSDMD‐FL in the down‐regulation of HOTAIR or miR‐22 mimic group were significantly lower than those in the high UA‐only group (Figure 5A). The release of the inflammatory cytokines IL‐1β and IL‐18 and LDH into the culture supernatant was also significantly lower in the down‐regulation of HOTAIR or miR‐22 mimic group than those in the high UA‐only group (Figure 5B,C), and cell proliferation significantly increased in the down‐regulation of HOTAIR or miR‐22 mimic group than those in the high UA‐only group (Figure 5D). However, the levels of these proteins were significantly higher in the suppression of miR‐22 and HOTAIR knockdown group than in the HOTAIR knockdown group (Figure 5A). The release of inflammatory cytokines IL‐1 and IL‐18 and LDH was also significantly higher in the suppression of miR‐22 and HOTAIR knockdown group than in the HOTAIR knockdown group (Figure 5E,F). Cell proliferation was significantly lower in the suppression of miR‐22 and HOTAIR knockdown group than in the HOTAIR knockdown group (Figure 5G).

### 3.6 HUA cause endothelial cell inflammation and pyroptosis by regulating the miR‐22/NLRP3 axis through HOTAIR

**FIGURE 6:**
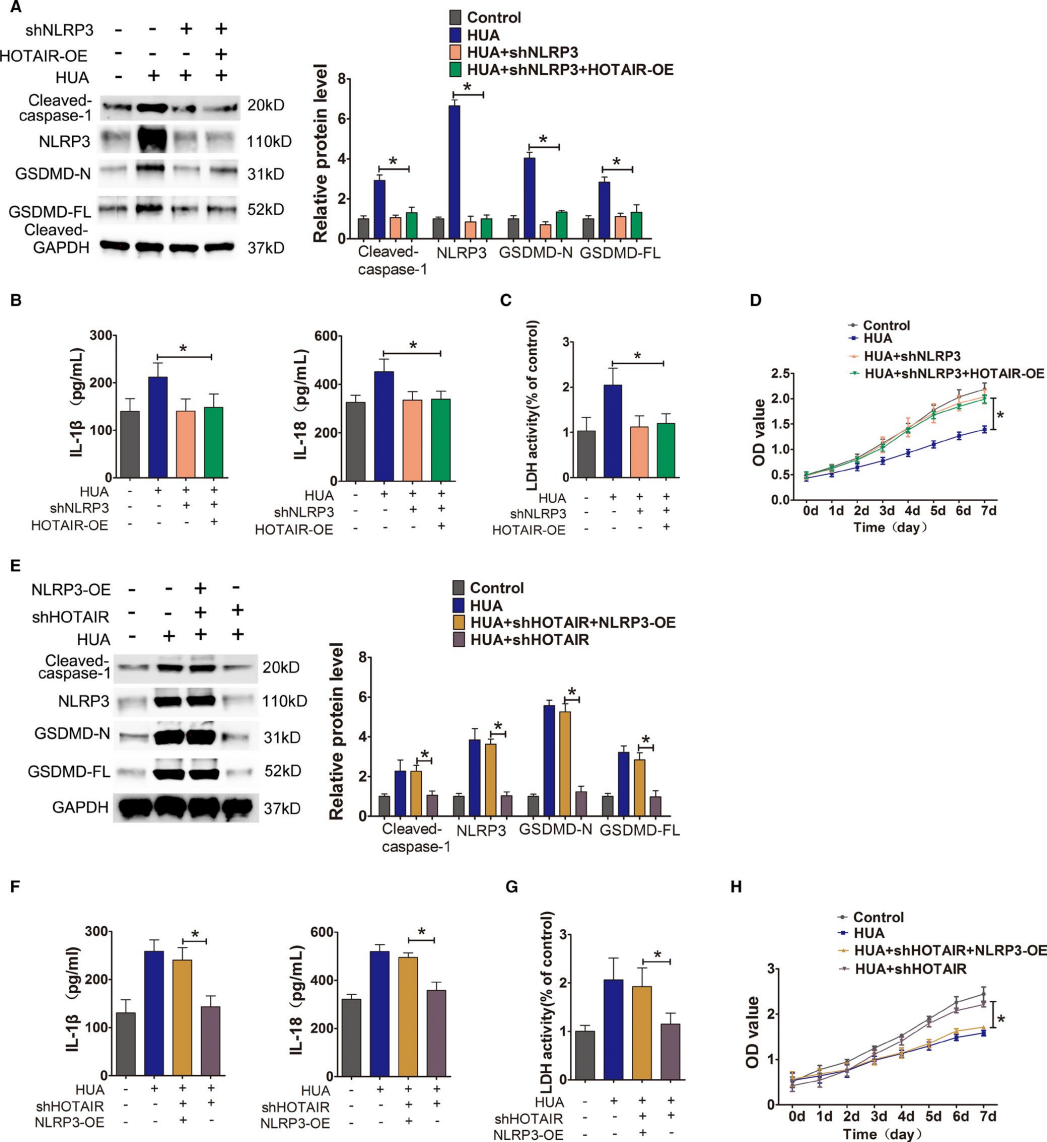
Identification of the role of NLRP3 in the HOTAIR‐induced pyroptosis pathway. (A) Protein levels of caspase‐1, NLRP3, GSDMD‐N and GSDMD‐FL, as measured by Western blot analysis; quantification normalized to GAPDH after shNLRP3 transfection. The expression of NLRP3, caspase‐1, GSDMD‐N and GSDMD‐FL decreased in HUA+shNLRP3+HOTAIR‐OE group; *p* < 0.05 compared to HUA group; *n* = 3 in each group. (B,C) Levels of IL‐1β, IL‐18 and LDH in cell culture supernatants, measured by ELISA after shNLRP3 transfection. The levels of IL‐1β, IL‐18 and LDH in culture supernatants decreased significantly in HUA+shNLRP3 + HOTAIR‐OE group; *p* < 0.05 compared to HUA group; *n* = 3 in each group. (D) Cell viability was determined using the CCK‐8 assay after shNLRP3 transfection. The proliferation of HUA+shNLRP3 + HOTAIR‐OE group significantly increased; *p* < 0.05 compared to HUA group; *n* = 3 in each group. (E) Protein levels of caspase‐1, NLRP3, GSDMD‐N and GSDMD‐FL, as measured by Western blot analysis; quantification normalized to GAPDH after HUVECs’ HOTAIR knockdown and NLRP3 overexpressing, followed by UA stimulation. The expression of NLRP3, caspase‐1, GSDMD‐N and GSDMD‐FL increased in UA+shHOTAIR+NLRP3‐OE group; *p < 0.05 compared to UA+shHOTAIR group; *n* = 3 in each group. (F,G) Levels of IL‐1β, IL‐18 and LDH in cell culture supernatants, measured by ELISA after HUVECs’ HOTAIR knockdown and NLRP3 overexpressing, followed by UA stimulation. The levels of IL‐1β, IL‐18 and LDH increased in UA+shHOTAIR+NLRP3‐OE group; *p < 0.05 compared to UA+shHOTAIR group; *n* = 3 in each group. (H) Cell viability was determined using the CCK‐8 assay after HUVECs’ HOTAIR knockdown and NLRP3 overexpressing, followed by UA stimulation. The proliferation of HUVECs in UA+shHOTAIR+NLRP3‐OE group significantly decreased. *p < 0.05 compared to UA+shHOTAIR group; *n* = 3 in each group

To further investigate whether HOTAIR induces inflammation and pyroptosis through the NLRP3 inflammasome, we used shRNA‐expressing lentiviruses to knock down NLRP3 expression in HOTAIR‐overexpressing endothelial cells, followed by HUA stimulation and culture. The results showed that after NLRP3 knockdown, the overexpression of HOTAIR did not induce the upregulation of pyroptosis‐associated proteins caspase‐1, GSDMD‐N and GSDMD‐FL, and the expression levels of these proteins were significantly lower than those in the HUA‐only treatment group (Figure 6A). Additionally, there were no significant differences in the release of the inflammatory cytokines IL‐1β and IL‐18 and LDH into the culture supernatant, but lower than those in the HUA‐only treatment group (Figure 6B,C), and there were no abnormal changes in cell proliferation (Figure 6D). Furthermore, we conducted an experiment to verify that whether upregulating NLRP3 expression could rescue the effect of silencing of HOTAIR on cell pyroptosis. We used shRNA‐expressing lentiviruses to knock down HOTAIR expression in NLRP3‐overexpressing endothelial cells, followed by UA stimulation and culture. The results showed that after HOTAIR knockdown, the overexpression of NLRP3 induced the upregulation of pyroptosis‐associated proteins, including NLRP3, caspase‐1, GSDMD‐N and GSDMD‐FL, and the expression levels of these proteins were significantly higher than those in the HOTAIR knockdown group (Figure 6E). Additionally. The secretion of the inflammatory cytokines IL‐1β and IL‐18 and LDH significantly increased (Figure 6F,G) in NLRP3‐overexpressing group, and cell proliferation also significantly inhibited in NLRP3‐overexpressing group (Figure 6H).

**FIGURE 7:**
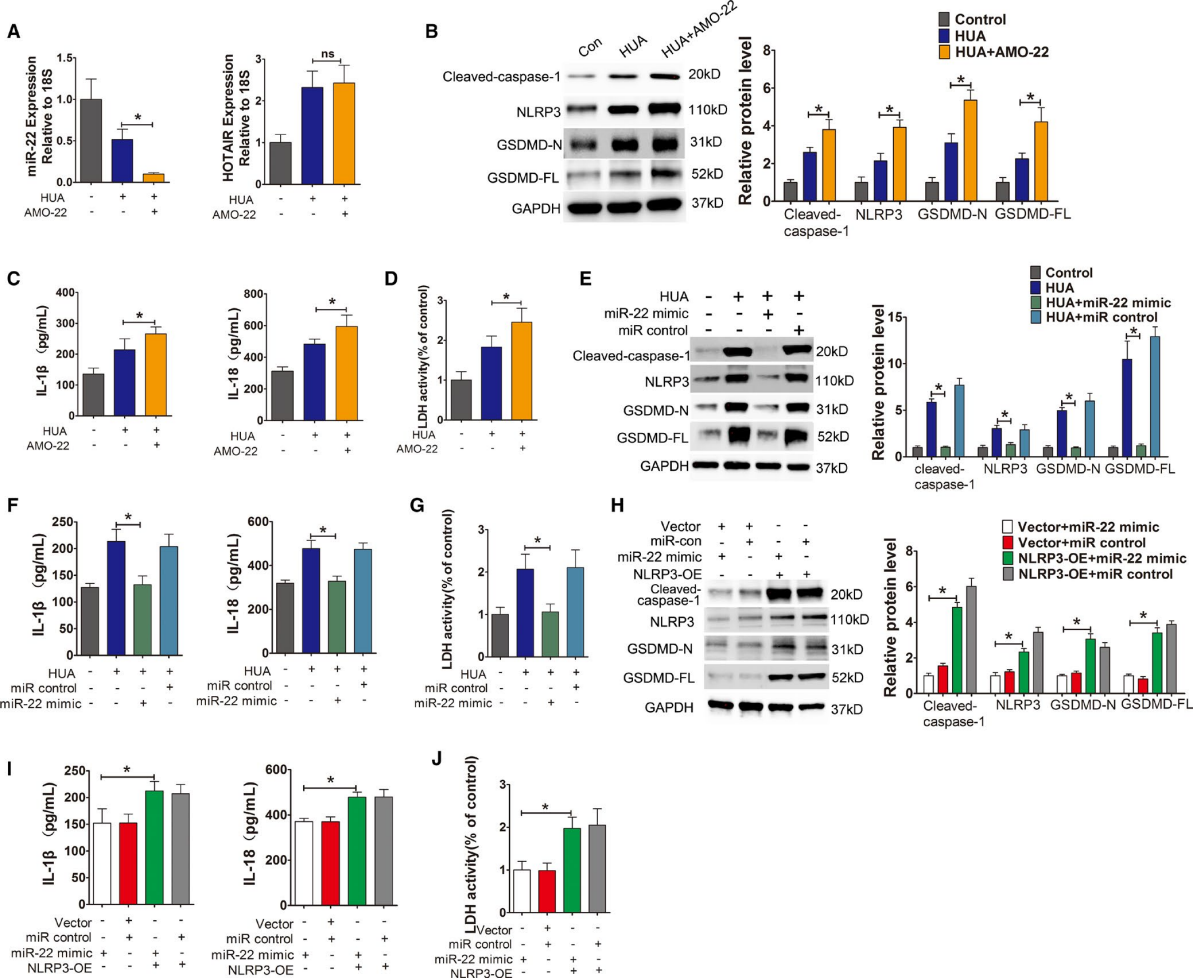
HUA promoted HUVECs inflammation and pyroptosis through miR‐22/NLRP3. (A) Relative HOTAIR expression was measured by qPCR after incubation with AMO‐22. The expression of HOTAIR did not change after incubation in HUA+AMO‐22 group. *p* > 0.05 compared to the HUA group; *n* = 3 in each group. (B) Protein levels of caspase‐1, NLRP3, GSDMD‐N and GSDMD‐FL, as measured by Western blot analysis; quantification normalized to GAPDH after incubation with AMO‐22. After knocking down miR‐22 with AMO‐22, the expression of NLRP3, caspase‐1, GSDMD‐N and GSDMD‐FL increased in HUA+AMO‐22 group; *p < 0.05 compared to the HUA group; *n* = 3 in each group. (C,D) Levels of IL‐1β, IL‐18 and LDH in cell culture supernatants, measured by ELISA after incubation with AMO‐22. The levels of IL‐1β, IL‐18 and LDH increased in HUA+AMO‐22 group; *p < 0.05 compared to the HUA group; *n* = 3 in each group. (E) Protein levels of caspase‐1, NLRP3, GSDMD‐N and GSDMD‐FL, as measured by Western blot analysis; quantification normalized to GAPDH after incubation with a miR‐22 mimic. After incubation with the miR‐22 mimic in a HUA environment, the expression of NLRP3, caspase‐1, GSDMD‐N and GSDMD‐FL significantly decreased; *p < 0.05 compared to the HUA group; *n* = 3 in each group. (F,G) Levels of IL‐1β, IL‐18 and LDH in cell culture supernatants, measured by ELISA after incubation with a miR‐22 mimic. The levels of IL‐1β, IL‐18 and LDH significantly decreased in HUA+miR‐22 mimic group; *p < 0.05 compared to the HUA group; *n* = 3 in each group. (H) Protein levels of caspase‐1, NLRP3, GSDMD‐N and GSDMD‐FL, as measured by Western blot analysis; quantification normalized to GAPDH in the NLRP3‐OE group after incubation with a miR‐22 mimic. In the NLRP3‐OE group after incubation with the miR‐22 mimic, the expression of NLRP3, caspase‐1, GSDMD‐N and GSDMD‐FL significantly increased in NLRP3‐OE+miR‐22 mimic group compared to that in the Vector +miR‐22 mimic group. *p < 0.05 compared to the Vector +miR‐22 mimic group; *n* = 3 in each group. (I,J) Levels of IL‐1β, IL‐18 and LDH in cell culture supernatants, measured by ELISA using the NLRP3‐OE group after incubation with a miR‐22 mimic. The levels of IL‐1β, IL‐18 and LDH were significantly increased in NLRP3‐OE+miR‐22 mimic group compared to those in the Vector +miR‐22 mimic group; *p < 0.05 compared to the vector +miR‐22 mimic group; *n* = 3 in each group

Next, we used AMO‐22 (miR‐22 inhibitor) to knock down miR‐22 in cells, which were then stimulated with HUA. The qPCR results showed that HOTAIR expression did not change significantly (Figure 7A). Compared with that in the HUA‐only group, the expression of the NLRP3 inflammasome, caspase‐1 and the pyroptosis‐related proteins GSDMD‐N and GSDMD‐FL increased (Figure 7B) and the secretion of the inflammatory cytokines IL‐1β and IL‐18 and LDH significantly increased (Figure 7C,D) in the AMO‐22‐treated cells.

In further experiments, the cells were stimulated with high levels of UA after transfection with a miR‐22 mimic. Compared with those in the control group, the expression levels of the NLRP3 inflammasome, caspase‐1 and the pyroptosis‐related proteins GSDMD‐N and GSDMD‐FL in the mimic group did not change significantly; however, the expression levels of those proteins were significantly lower in the mimic group than in the HUA‐only group (Figure 7E). The release of the inflammatory cytokines IL‐1β and IL‐18 and LDH into the culture supernatant was also significantly lower in the mimic group than in the high UA‐only group (Figure 7F,G).

Interestingly, the above phenomena were not observed when the miR‐22 mimic was added to the NLRP3 overexpression group. Expression levels of the pyroptosis‐related proteins NLRP3, caspase‐1, GSDMD‐N and GSDMD‐FL were detected by Western blot, and the levels of these proteins were significantly higher in the NLRP3 overexpression group than in the no‐load vector group (Figure 7H). The release of inflammatory cytokines IL‐1 and IL‐18 and LDH was also significantly higher in the NLRP3 overexpression group than in the no‐load vector group (Figure 7I,J).

### 3.7 Inhibition of HOTAIR attenuates inflammatory kidney injury in hyperuricaemia mice

**FIGURE 8:**
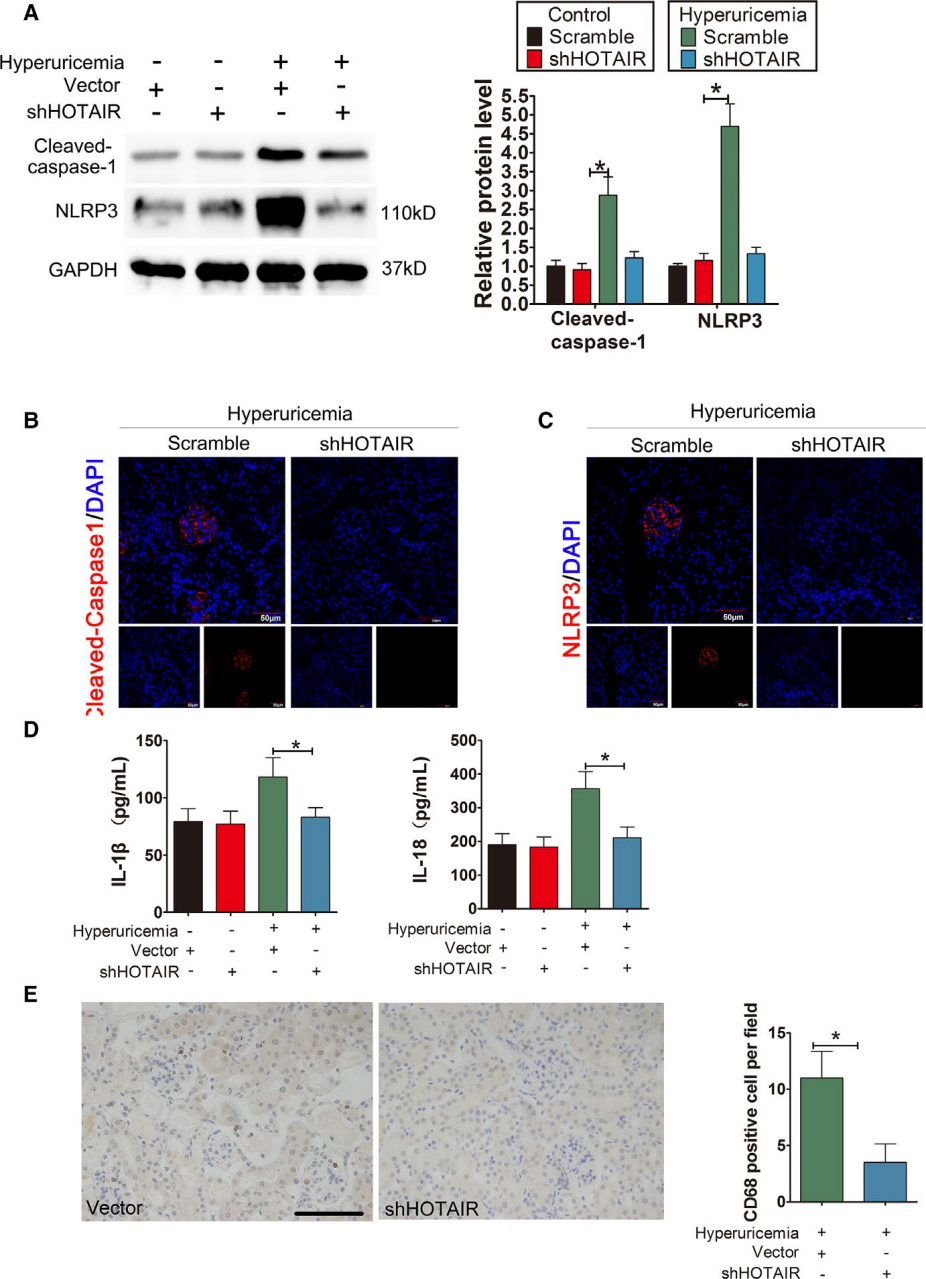
Knockdown of HOTAIR ameliorates renal inflammation in HUA mice. (A) The protein levels of caspase‐1, NLRP3, GSDMD‐N and GSDMD‐FL, as measured by Western blot analysis; quantification normalized to GAPDH in renal tissue from hyperuricaemia mice after shRNA treatment. The protein levels of caspase‐1, NLRP decreased remarkably in hyperuricaemia+shHOTAIR group; *p < 0.05 compared to the hyperuricaemia +vector group; *n* = 6 in each group. (B,C) Immunofluorescence images showing the expression of caspase‐1, NLRP3 in renal tissue from hyperuricaemia mice after shRNA treatment. Tissue immunofluorescence showed that the fluorescence intensity of glomerular NLRP3 and caspase‐1 in the shRNA‐treated group was significantly lower than that in the hyperuricaemia+shHOTAIR group; *p < 0.05 compared to the control group; *n* = 6 in each group (scale bar, 50 μm; magnification, 400×); blue: nuclear staining (DAPI), red: caspase‐1 and NLRP3 staining. (D) The serum levels of IL‐1β and IL‐18, measured by ELISA. The serum levels of IL‐1β and IL‐18 decreased remarkably in the hyperuricaemia+shHOTAIR group; *p < 0.05 compared to the in the hyperuricaemia+Vector group; *n* = 6 in each group. (E) CD68 immunohistochemistry of the renal interstitium and glomerular mesangial area in the hyperuricaemia+vector group and hyperuricaemia+shHOTAIR group. Immunohistochemistry of CD68 in renal tissue from hyperuricaemia mice after shRNA treatment. Compared with that in the hyperuricaemia+Vector group, the down‐regulation of HOTAIR by shHOTAIR significantly reduced the infiltration of positive CD68 macrophages in the renal interstitium and glomerular Mesangial area in hyperuricaemia+shHOTAIR group; ∗*p* < 0.05 compared to hyperuricaemia+vector group; *n* = 6 in each group (scale bar, 100 μm; magnification, 400×)

To explore novel strategies for the targeted treatment of UA nephropathy, based on the above studies, we injected shHOTAIR lentiviruses, via tail vein, into hyperuricaemia mice and subsequently assessed the inflammation status in kidney tissues The results showed that the protein expression levels of NLRP3 and caspase‐1 in the renal tissues of the treatment group were significantly lower than those in the unrelated lentivirus (scramble) group (Figure 8A). The immunofluorescence intensity of NLRP3 and caspase‐1 in kidney tissue from the treatment group was significantly lower than that in kidney tissue from the model group (Figure 8B,C). Furthermore, in the treatment group, serum inflammatory cytokines IL‐1β and IL‐18 decreased, with a statistically significant difference compared with the control group (Figure 8D). Further immunohistochemical analysis of kidney sections revealed that compared with the model‐only group, the use of shHOTAIR lentivirus to down‐regulate HOTAIR significantly reduced the infiltration of CD68+ macrophages around the blood vessels of the glomerular mesangial area and renal interstitium (Figure 8E).

## 4 DISCUSSION

Studies have confirmed that HOTAIR plays a role in diabetic retinopathy and leads to diabetic retinal endothelial cell dysfunction.^33^ Related studies have also shown that HOTAIR is highly expressed in patients with diabetic nephropathy and in the kidney tissues of animals.^34^ However, the role and mechanism of HOTAIR in HUA‐induced endothelial injury are not fully understood. In this study, we found that the expression level of HOTAIR in hyperuricaemia patients was significantly higher than that in the normal population. The same results were also observed in a hyperuricaemia mouse model and in HUVECs stimulated with HUA. lncRNAs can function as endogenous competitive RNAs of miRNAs to regulate miRNA expression and biological gene functions by sequence complementarity.^35^, ^36^ Therefore, we hypothesized that HOTAIR plays a similar role in HUA. Then, what is its target miRNA? Using bioinformatics prediction tools, we determined that miR‐22 has a HOTAIR binding site; a dual‐luciferase reporter assay confirmed that miR‐22 can bind to HOTAIR, a result that is consistent with literature reports.^31^ miR‐22 is closely associated with a variety of diseases, including tumours and cardiovascular diseases.^37^, ^38^ In sepsis‐induced acute kidney injury, HOTAIR can act as a molecular sponge to adsorb miR‐22, causing renal tubular epithelial cell apoptosis.^31^ However, there is currently no evidence supporting the role of miR‐22 in HUA‐induced kidney injury. We observed a decrease in serum miR‐22 expression in hyperuricaemia patients and a negative correlation between miR‐22 and HOTAIR expression. In in vitro experiments, HOTAIR overexpression in endothelial cells significantly reduced miR‐22 expression and increased the secretion of inflammatory cytokines; these effects were offset by a miR‐22 mimic. In contrast, after HOTAIR knockdown and HUA stimulation, the expression levels of miR‐22 and inflammatory cytokines were not significantly different from those in the normal control group. RNA pulldown assay result showed that HOTAIR could also pull down miR‐22 by using a biotin‐labelled specific HOTAIR probe through a pulldown system, indicating a sequence‐specific recognition between miR‐22 and HOTAIR.RIP experiments suggested that Ago2 immunoprecipitated with a higher amount of HOTAIR and miR‐22 than did IgG. Therefore, the competitive binding between HOTAIR and miR‐22 transcripts is based on forming a RISC with Ago2, and HOTAIR might promote HUA‐induced inflammatory kidney injury by competitively binding miR‐22.

Actually, lncRNAs can regulate gene expression at a variety of levels.^39^ In addition to acting as molecular sponge to bind miRNAs and prevent miRNAs from binding to their target mRNAs,^36^ lncRNAs can also regulate gene expression as epigenetic regulators. lncRNAs could also regulate gene expression through epigenetic modification. LncRNAs can interact with chromatin and then recruit protein complexes to remodel chromatin states and change DNA/RNA methylation status, thus regulating gene expression.^40^ NEAT1, a cancer lncRNA, controls 13.3% of genes in the PI3K‐AKT signalling pathway by interacting with distal regulatory elements.^41^ H19 binds EZH2 in glioblastoma cells, and that EZH2 binding to NKD1 and other promoters is impaired by H19 silencing.^42^ Therefore, it is worth further exploring whether epigenetic regulation plays a role in HOTAIR‐induced endothelial injury in HUA.

Numerous studies have confirmed that inflammatory responses are closely associated with the occurrence and development of hyperuricaemia.^4^, ^7^, ^8^ As a newly discovered mode of programmed cell death, pyroptosis promotes the release of inflammatory cytokines and accelerates disease progression. Studies have shown that pyroptosis is closely related to the occurrence and development of UA nephropathy.^43^ The canonical pyroptosis pathway relies on caspase‐1 activation.^44^ Through the sensing of pathogen‐related molecular patterns and/or damage‐associated molecular patterns, including influenza viruses, listeria monocytogenes, extracellular ATP, hyaluronic acid, glucose, amyloid‐β, silicon, uric acid crystals and cholesterol crystals, intracellular pattern recognition receptors activate the NLRP3 inflammasome to activate caspase‐1. Activated caspase‐1, on the one hand, cleaves the gasdermin D protein to form a peptide segment containing the active N‐terminal domain of gasdermin D, which induces cell membrane perforation, cell rupture and content release and causes pyroptosis.^45^, ^46^ On the other hand, activated caspase‐1 cleaves IL‐1β and IL‐18 precursors to form active IL‐1β and IL‐18, which are released extracellularly to recruit inflammatory cells that aggregate during the inflammatory response.^45^ High level of UA in the blood leads to the formation of urate crystal, which would deposit in the renal interstitium and lead to inflammation. UA, as a damage‐associated molecular pattern, can activate the NLRP3 inflammasome in macrophages, which in turn leads to pyrotosis.^47^ We found that in HUA‐treated HUVECs, the NLRP3 inflammasome was activated, followed by pyroptosis and an inflammatory response. An increase in CD68+ cell infiltration was also observed in hyperuricaemia mice, suggesting inflammatory injury in the renal microcirculation and endothelium in this model and the release of inflammatory factors, the infiltration of chemotactic inflammatory cells and the aggravation of kidney damage. Notably, in the present study, gain‐ or loss‐of‐function experiments indicated that NLRP3 overexpression induced an inflammatory response and pyroptosis in HUVECs, whereas the pre‐treatment of HUVECs with shNLRP3 to knock down NLRP3 gene expression followed by UA stimulation had opposite effects. These results suggest that the NLRP3 inflammasome plays an important role in the pathogenesis of HUA‐induced renal injury.

How does HOTAIR, which is highly expressed in endothelial cells in a HUA environment, mediate NLRP3 activation and pyroptosis? Bioinformatic analysis indicated that miR‐22, which binds to HOTAIR, can also bind to the 3′‐UTR region of NLRP3. A dual‐luciferase reporter assay also confirmed this result. Subsequent in vivo and in vitro experiments further demonstrated that high levels of HOTAIR expression and decreased miR‐22 expression occurred in a HUA environment, whereas the expression levels of pyroptosis‐related proteins (caspase‐1, NLRP3, GSDMD‐N and GSDMD‐FL) were elevated, LDH release was elevated, and cell proliferation was significantly inhibited, demonstrating the occurrence of pyroptosis. If HOTAIR expression in endothelial cells was knocked down or a miR‐22 mimic was used, the opposite effect was achieved. These results suggested that HOTAIR regulates the expression of NLRP3 by competitively binding to miR‐22, thereby inducing inflammation and pyroptosis. This regulatory mechanism of lncRNAs on pyroptosis is supported by the results of some studies. HOTAIR activates NLRP3‐mediated pyroptosis in SH‐SY5Y cells by negatively regulating miR‐326, thereby promoting neuronal injury in Parkinson's disease.^48^ In calcium oxalate–induced kidney stones, lncRNA‐00339 promotes renal tubular epithelial cell pyroptosis by regulating the miR‐22‐3p/NLRP3 axis.^49^

We examined the inhibitory effect on endothelial cell inflammation and pyroptosis in HUVEC through down‐regulation of HOTAIR. Compared to UA‐only group, we observed that the expression levels of pyroptosis‐related proteins were decreased, inflammatory cytokines and LDH release was decreased, and cell proliferation was significantly increased. We observed the same result when miR‐22 was overexpressed. What is more, suppression of miR‐22 restrained the inhibitory effects of HOTAIR knockdown on the inflammatory response and pyroptosis. We also observed that after the inhibition of miR‐22 by AMO‐22 in a HUA environment, the release of pyroptosis‐related proteins, inflammatory cytokines and LDH further increased compared to that in the HUA‐alone group; notably, the expression of HOTAIR was not affected by AMO‐22. The results further suggest that miR‐22 is a downstream target of HOTAIR. In addition, a miR‐22 mimic significantly reduced the expression of NLRP3 and other pyroptosis‐related proteins (caspase‐1, GSDMD‐N and GSDMD‐FL) in a HUA environment; furthermore, the release of inflammatory cytokines and LDH was also significantly reduced. However, the miR‐22 mimic could not reverse the inflammatory response and pyroptosis caused by NLRP3 overexpression, indicating that NLRP3 is a downstream target of miR‐22. Therefore, we infer that lncRNA‐HOTAIR induces pyroptosis of endothelial cells in a HUA environment through the miR‐22/NLRP3 axis and induces inflammatory injury.

Currently, the treatments for HUA are primarily drugs that inhibit UA production and promote UA excretion, while simultaneously improving quality of life.^50^, ^51^ Although some studies have developed inhibitors that target inflammasomes,^52^, ^53^ there is no report showing effective treatment for HUA‐induced inflammatory renal injury. Through tail vein injection of a shHOTAIR lentivirus, we attempted targeted gene therapy for hyperuricaemia mice. We found that shHOTAIR significantly improved the hyperuricaemia induced inflammatory state of the kidney, providing a preliminarily basis for the feasibility of gene‐targeted therapy for UA nephropathy.

A recent study deserves our attention. Battistelli et al focused on a strategy to counteract HOTAIR activity which is worth further exploring.^54^ In their study, they validated for the first time a dominant negative‐based RNA strategy to interfere with a pivotal function of a lncRNA. They designed a HOTAIR deletion mutant form (called HOTAIR‐sbid) to correspond to the putative Snail‐binding and depleted of the EZH2‐binding domain. HOTAIR‐sbid acted as a dominant negative of the endogenous HOTAIR. In both murine and human tumour cells, HOTAIR‐sbid impaired the ability of HOTAIR to bind Snail and, in turn, trigger H3K27me3/EZH2‐mediated repression of Snail epithelial target genes. HOTAIR‐sbid was proven to reduce cellular motility, invasiveness, anchorage‐independent growth and not impair the TGF‐ mediated mesenchymal gene expression. They provide evidence on a lncRNA‐based strategy to treat diseases caused by abnormal expression of LncRNAs. We will apply this strategy in our research in the future.

In summary, this study elucidated the mechanism underlying the promotion of inflammatory renal injury by HOTAIR in a HUA environment. As a ceRNA of miR‐22, HOTAIR promotes the expression of the NLRP3 inflammasome in endothelial cells and induces inflammation and pyroptosis. Therefore, HOTAIR is proposed as a suitable therapeutic target for UA nephropathy, providing a novel theoretical basis for developing new drugs and correcting endothelial dysfunction in a HUA environment.

## CONFLICT OF INTEREST

The authors declare that they have no conflict of interest.

## AUTHOR CONTRIBUTION

**Kun Chi:** Writing‐original draft (equal). **Xiaodong Geng:** Writing‐original draft (equal). **Chao Liu:** Data curation (equal). **Yang Zhang:** Formal analysis (equal). **Jie Cui:** Formal analysis (equal). **GuangYan Cai:** Data curation (equal). **Xiangmei Chen:** Formal analysis (equal). **Fangfang Wang:** Formal analysis (equal). **Quan Hong:** Writing‐original draft (equal).

## Data Availability

The data that support the findings of this study are available from the corresponding author upon reasonable request.
