#  LncRNA‐HOTAIR promotes endothelial cell pyroptosis by regulating the miR‐22/NLRP3 axis in hyperuricemia

**DOI:** 10.1111/jcmm.17777

**Published:** 2023-07-02

In Chi et al.,^1^ the published article contains errors in Figures 3 and 8. The bar‐graph (Figure 3F) was accidentally misused as Figure 3G. The immunofluorescence picture of the control group (Figure 2G) was accidentally misused in shHOTAIR group (Figure 8B). The corrected figures and their legends are shown below. The authors confirmed all results and conclusions of this article remain unchanged.

**FIGURE 3:**
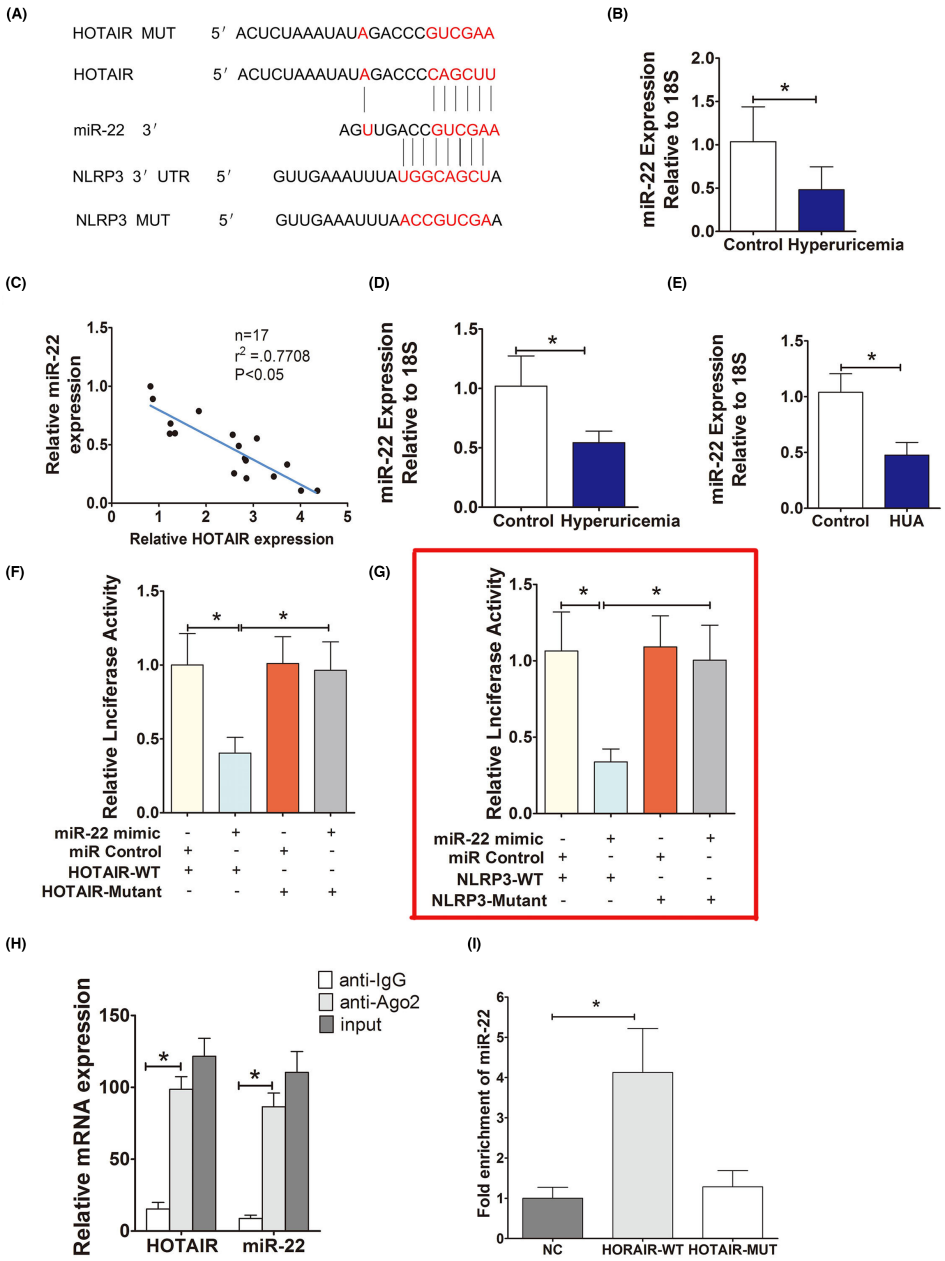
HOTAIR, as a ceRNA, regulates the expression of miR‐22. (A) The sequences of HOTAIR and NLRP3 aligned with miR‐22, including the wildtype (WT) and a mutant. Schematic illustration of the presumed target site for HOTAIR and NLRP3 in miR‐22. (B) Serum levels of miR‐22 in normal controls and hyperuricemia patients, measured by qPCR. The expression of miR‐22 in hyperuricemia group was significantly downregulated and negatively correlated with the expression of HOTAIR; *p < 0.05 compared to the control group; *n* = 17 in each group. (C) Correlation analysis between HOTAIR and miR‐22 levels in normal controls and hyperuricemia patients. miR‐22 was negatively correlated with HOTAIR (*r* ^2^ = −0.77); *p < 0.05 compared to the normal controls; *n* = 17 in each group. (D, E) The levels of miR‐22 in hyperuricemia mice (*n* = 6) and HUVECs (*n* = 3), measured by qPCR. The expression of miR‐22 in HUA mice and HUA‐stimulated HUVECs was significantly downregulated; *p < 0.05 compared to the control group; *n* = 6 in each group. (F) Luciferase activity results. There is direct binding between HOTAIR and miR‐22; *p < 0.05 versus the mimic NC + HOTAIR‐WT group; *n* = 3 in each group. (G) Luciferase activity results. miR‐22 directly regulates the expression of NLRP3, *p < 0.05 compared to the mimic NC + NLRP3‐WT group; *n* = 3 in each group. (H) RIP assays using cell lysate IgG or anti‐Ago2 as the input. Relative expression levels of HOTAIR and miR‐22 in HUVECs were detected by qPCR and normalized to 18 s. The results indicated higher HOTAIR and miR‐22 RNA levels in Ago2 immunoprecipitates relative to control IgG immunoprecipitates; *p < 0.05 compared to the anti‐IgG group; *n* = 3 in each group. (I) Detections of miR‐22 using qRT‐PCR in the same sample pulled down by biotinylated HOTAIR and NC probe. miR‐22 expression was significantly higher in the HOTAIR‐WT group; *p < 0.05 compared to NC group; *n* = 3 in each group.

**FIGURE 8:**
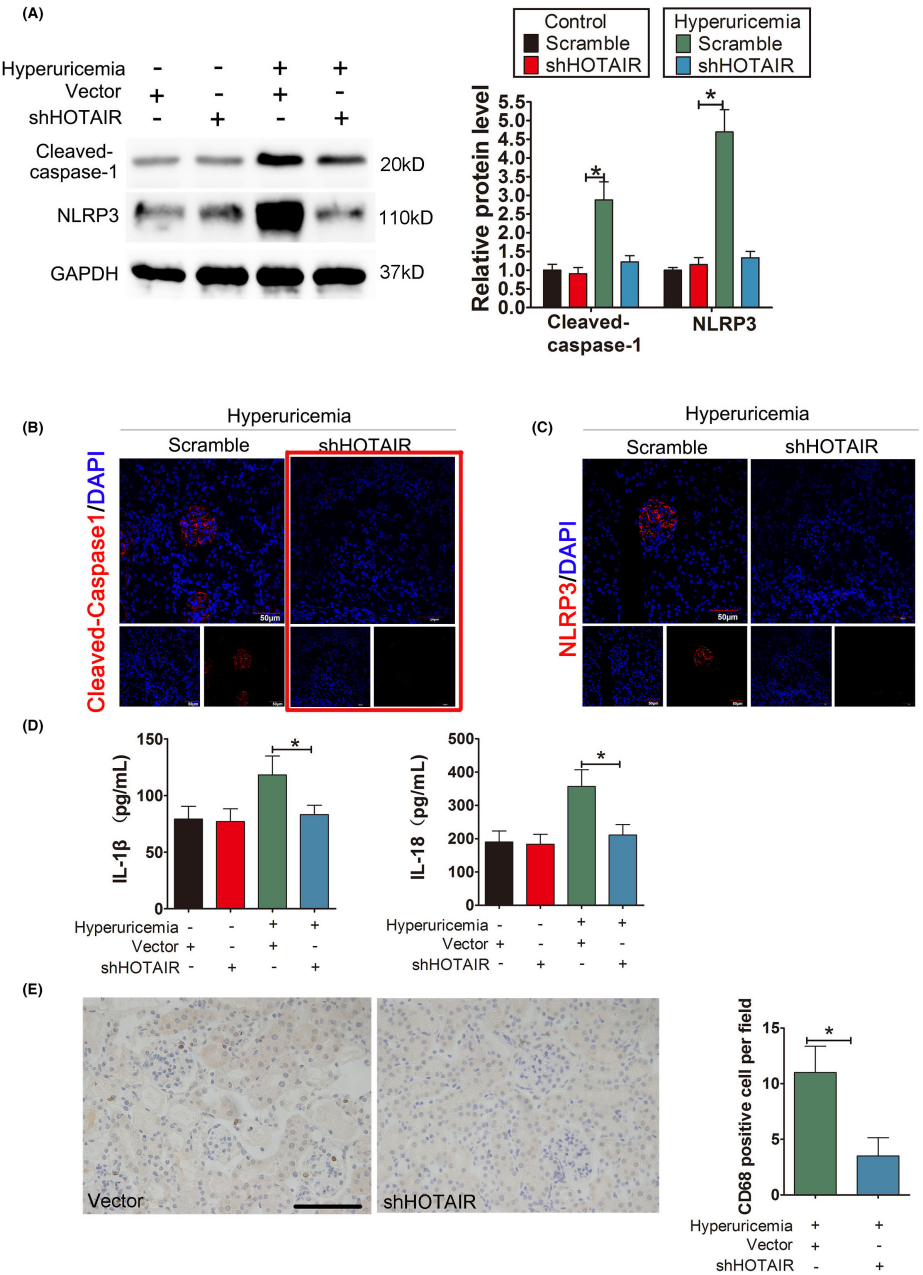
Knockdown of HOTAIR ameliorates renal inflammation in HUA mice. (A) The protein levels of caspase‐1, NLRP3, GSDMD‐N and GSDMD‐FL, as measured by Western blot analysis; quantification normalized to GAPDH in renal tissue from hyperuricemia mice after shRNA treatment. The protein levels of caspase‐1, NLRP decreased remarkably in hyperuricemia + shHOTAIR group; *p < 0.05 compared to the hyperuricemia + vector group; *n* = 6 in each group. (B, C) Immunofluorescence images showing the expression of caspase‐1, NLRP3 in renal tissue from hyperuricemia mice after shRNA treatment. Tissue immunofluorescence showed that the fluorescence intensity of glomerular caspase‐1 and NLRP3 in the shRNA‐treated group was significantly lower than that in the hyperuricemia + shHOTAIR group; *p < 0.05 compared to the control group; *n* = 6 in each group (scale bar, 50 μm; magnification, 400×); blue: nuclear staining (DAPI), red: caspase‐1 and NLRP3 staining. (D) The serum levels of IL‐1β and IL‐18, measured by ELISA. The serum levels of IL‐1β and IL‐18 decreased remarkably in the hyperuricemia + shHOTAIR group; *p < 0.05 compared to the in the hyperuricemia + Vector group; *n* = 6 in each group. (E) CD68 immunohistochemistry of the renal interstitium and glomerular mesangial area in the Hyperuricemia + vector group and hyperuricemia + shHOTAIR group. Immunohistochemistry of CD68 in renal tissue from hyperuricemia mice after shRNA treatment. Compared with that in the hyperuricemia + Vector group, the downregulation of HOTAIR by shHOTAIR significantly reduced the infiltration of positive CD68 macrophages in the renal interstitium and glomerular Mesangial area in hyperuricemia + shHOTAIR group; *p < 0.05 compared to hyperuricemia + vector group; *n* = 6 in each group(scale bar, 100 μm; magnification, 400×).
